# Arsenic Removal Using Unconventional Material with Iron Content: Batch Adsorption and Column Study

**DOI:** 10.3390/toxics11100849

**Published:** 2023-10-10

**Authors:** Cosmin Vancea, Georgiana Mladin, Mihaela Ciopec, Adina Negrea, Narcis Duteanu, Petru Negrea, Giannin Mosoarca, Catalin Ianasi

**Affiliations:** 1 Faculty of Industrial Chemistry and Environmental Engineering, Politehnica University Timisoara, Bd. V. Parvan, No. 6, 300223 Timisoara, Romania; cosmin.vancea@upt.ro (C.V.); georgiana.mladin@student.upt.ro (G.M.); adina.negrea@upt.ro (A.N.); narcis.duteanu@upt.ro (N.D.); petru.negrea@upt.ro (P.N.); 2 “Coriolan Drăgulescu” Institute of Chemistry, Bv. Mihai Viteazul, No. 24, 300223 Timisoara, Romania; ianasic@acad-icht.tm.edu.ro

**Keywords:** arsenic removal, unconventional material with iron content (UMIC), batch absorption, fixed-bed column adsorption, mechanism of adsorption

## Abstract

The remediation of arsenic contamination in potable water is an important and urgent concern, necessitating immediate attention. With this objective in mind, the present study investigated arsenic removal from water using batch adsorption and fixed-bed column techniques. The material employed in this study was a waste product derived from the treatment of groundwater water for potable purposes, having a substantial iron composition. The material’s properties were characterized using scanning electron microscopy (SEM), energy-dispersive X-ray spectroscopy (EDX), and Fourier-transformed infrared spectroscopy (FT-IR). The point of zero charge (pH_PZC_) was measured, and the pore size and specific surface area were determined using the BET method. Under static conditions, kinetic, thermodynamic, and equilibrium studies were carried out to explore the influencing factors on the adsorption process, namely the pH, contact time, temperature, and initial arsenic concentration in the solution. It was found that the adsorption process is spontaneous, endothermic, and of a physical nature. In the batch adsorption studies, the maximum removal percentage was 80.4% after 90 min, and in a dynamic regime in the fixed-bed column, the efficiency was 99.99% at a sludge:sand = 1:1 ratio for 380 min for a volume of water with arsenic of ~3000 mL. The kinetics of the adsorption process conformed to a pseudo-second-order model. In terms of the equilibrium studies, the Sips model yielded the most accurate representation of the data, revealing a maximum equilibrium capacity of 70.1 mg As(V)/g sludge. For the dynamic regime, the experimental data were fitted using the Bohart–Adams, Thomas, and Clark models, in order to establish the mechanism of the process. Additionally, desorption studies were conducted, serving as an essential step in validating the practical applicability of the adsorption process, specifically in relation to the reutilization of the adsorbent material.

## 1. Introduction

The protection of the environment assumes a position of major significance, as it encompasses the preservation of ecological equilibrium, the enhancement of natural elements’ quality, and the assurance of suitable habitation and labor conditions for present and forthcoming generations. The pervasive degradation of the environment in specific regions across the globe, coupled with the depletion of non-renewable resources, remains a perpetual concern for environmentalists striving to uphold environmental quality [1,2].

Arsenic is an element that permeates the environment through a variety of natural and anthropogenic sources. The arsenic contamination of potable water has emerged as a widespread issue, focusing international attention in recent times. The levels of arsenic in rivers, lakes, and groundwater exhibit a broad spectrum of concentrations, as documented by different researchers, yielding contradictory findings. Nevertheless, in contaminated water, the arsenic content can vary from hundreds to thousands of micrograms per liter, starkly surpassing the maximum permissible threshold of 10 micrograms per liter, as stipulated by the World Health Organization [3,4,5,6].

The arsenic contamination of drinking water gives rise to major problems due to arsenic poisoning. Chronic exposure to arsenic includes cutaneous diseases (pigmentation, skin cancer), as well as a variety of maladies impacting the cardiovascular, neurological, hematological, renal, and respiratory systems, in addition to a heightened risk of developing lung, bladder, liver, kidney, and prostate cancer. The populations most profoundly affected by arsenic contamination predominantly hail from economically disadvantaged backgrounds or marginalized communities [7].

The perils associated with prolonged exposure to arsenic are widely recognized, with the afflicted populace enduring a multitude of health problems that manifest not only as dermatological disorders, but also implicating vital internal organs such as the bladder, kidneys, and lungs, while further encompassing cardiovascular complications, respiratory ailments, diabetes, and other related conditions [5,8].

Underground sources of potable water are widely recognized as a primary contributor responsible for the chronic health problems related to arsenic poisoning of the population worldwide. Arsenic in drinking water sources is primarily found as As(V) and more frequently as As(III), with the latter being even more toxic [4,7,8,9,10,11].

Various techniques have been developed to mitigate arsenic contamination in water sources: precipitation–filtration processes [3,12], coagulation–precipitation using aluminum salts, iron, and/or lime water as coagulation agents [12], combined processes such as photocatalysis coupled with complexation and filtration [4,13], aeration, chemical oxidation, oxidation–coagulation, or oxidation–precipitation [14,15], electro-coagulation [14], membrane separation techniques like nanofiltration, reverse osmosis, and electrodialysis [3], ion exchange [16], and adsorption [3,4,5,17].

However, techniques based on reverse osmosis and electrodialysis [5] are generally expensive and exhibit limited efficacy if the oxidation of As(III) to As(V) is not executed beforehand, presenting a superior effectiveness in removing As(V) compared to As(III) [4]. Other techniques such as solvent extraction or bioremediation [4,18] are encumbered by their high costs, and are not well-suited for small-scale water treatment systems.

The mechanism underlying the process of removing arsenic from water by adsorption depends primarily on the nature of the adsorbent material. The utilization of selective adsorption using a diverse range of substances, including biological materials, mineral oxides, activated carbon, waste and industrial by-products, and polymeric resins, has garnered considerable attention due to the promising outcomes achieved [4].

The arsenic removal mechanisms using iron compounds involve its adsorption onto the surface of the adsorbent, entrapment and binding of the arsenic within hydroxide flocs, and the formation of ferric arsenate (FeAsO_4_) through oxidation–reduction reactions [3,4,5].

Illustrative examples of low-cost, biomass and sludge adsorbents include agricultural waste or by-products [19,20], industrial sludges containing metal oxides/hydroxides [1,2,3], hydroxides, hydroxo-oxides, or iron oxides [4], hydrotalcites [4], algae, fungi, various bacteria [18], cellulose [4], and even human hair [21], which have proven their viability. The literature reports several iron-containing adsorbents, but their arsenic adsorption capacities are relatively low: 0.67 mg/g for iron oxide coated cement [22] and 10.6 mg/g for iron oxide-coated sand [23].

In this context, the objective of this study centered on the static and dynamic adsorption of As(V) using an unconventional material with iron content (UMIC) derived from groundwater treatment for drinking purposes; the process is affordable and eco-friendly, with a high iron content, rendering it ideal for As(V) removal.

## 2. Materials and Methods

### 2.1. Unconventional Material (UMIC) Characterization

The material used for the studies in static and dynamic modes in a fixed-bed column was the sludge obtained from a local groundwater treatment plant for drinking purposes. The sludge was dried at room temperature (25 °C), milled, and sieved, and the fraction under 1.25 mm was used without any other chemical treatment.

To ascertain its chemical composition, a sample comprising 3 g of the sludge was used. The sample underwent extraction with vigorous stirring, utilizing 21 mL of concentrated hydrochloric acid (37 wt.%, Sigma Aldrich, St. Louis, MO, USA) and 7 mL of nitric acid (63.013 wt.%, Sigma Aldrich, St. Louis, MO, USA), for 16 h at room temperature, followed by boiling under reflux for 2 h. The extract was then clarified and brought to the required volume with nitric acid. The contents of iron, manganese, calcium, magnesium, sodium, and potassium in the extract were determined via atomic absorption using a Varian SPECTRAA FS 280 atomic absorption spectrophotometer (Varian Inc., Mulgrave, Australia).

The material was characterized using scanning electron microscopy coupled with energy dispersive X-ray spectroscopy (SEM–EDX), using an X-ray energy dispersive spectrometer, FEI Quanta FEG 250 instrument (FEI, Eindhoven, The Netherlands), to establish the morphology and the predominant elements present in the material composition.

The presence of specific peaks related to the functional groups present in the material structure were studied using FT-IR spectroscopy with a Bruker Platinum ATR-QL Diamond FT-IR spectrometer (Bruker Optik GmbH, Ettlingen, Germany) in the range of 4000–400 cm^−1^.

The material pore size and the specific surface area were determined using the BET method (Brunauer, Emmett, Teller), using a Nova 1200e Quantachrome apparatus (Anton Paar GmbH, Graz, Austria).

The point of zero charge was determined using the method of setting the studied system to equilibrium [24,25]. For this study, 0.1 g of material was mixed with 25 mL of KCl 0.1 N solution, and then stirred with 200 rotations/minute at 298 K using a Julabo SW23 type thermostatic water bath (Julabo GmbH, Seelbach, Germany). The pHs of the KCl solutions were adjusted in a range of 2–12 using NaOH solutions in a concentration range between 0.05 N and 2 N, or HNO_3_ solutions with a concentration between 0.05 N and 2 N. The samples were filtered, and later the pH of the resulting solution was determined using a Mettler-Toledo, SevenCompact, S 210 pH meter (Mettler-Toledo, Columbus, OH, USA).

### 2.2. Batch Adsorption Study

The static adsorption mechanism was proposed based on kinetic, thermodynamic, and equilibrium studies, varying the contact time, temperature, and initial concentration of As(V). Simultaneously, the dynamic mode mechanism was studied through the examination of variations in the height of the fixed-bed column and the time evolution of the material quantity employed. Adsorption experiments were performed using three independent replicates.

In this research, the influence of pH on the adsorption process of As(V) (As_2_O_3_, Fluka, pa, ≥99.5%, Honeywell Research Chemicals, Seelze, Germany) on the material was studied, varying the pH in a range of 1–10, at an initial concentration of As(V) of *C*_0_ = 100 µg/L, for 0.1 g of material, 25 mL of solution, a contact time of 60 min, and a temperature of 298 K.

For the influence of contact time and temperature on the adsorption capacity of the studied material, 0.1 g of adsorbent and 25 mL of As(V) solution with a concentration of *C*_0_ = 100 µg/L were used. The samples were stirred at 200 rpm for different times (15, 30, 45, 60, 90, and 120 min) in a Julabo SW23 type water bath (Julabo GmbH, Seelbach, Germany) with a thermostat, and agitated at different temperatures (298 K, 308 K, 318 K, and 328 K).

In order to establish the effect of the initial concentration of As(V) on the adsorption capacity of the material, As(V) solutions with concentrations of 50, 75, 100, 150, 200, 250, 300, 350, and 500 mg/L were prepared. The adsorption was carried out at pH = 7–8, for 90 min and at a temperature of 298 K. The residual concentration of As(V) was measured using an atomic AA 6800, Schimadzu absorption spectrophotometer (Shimadzu Corporation, Kyoto, Japan) equipped with a graphite furnace.

To explore the kinetics governing the adsorption process of As(V) onto the material, the acquired experimental data were subjected to modeling, utilizing the pseudo-first-order and pseudo-second-order equations shown in Table 1.

Thermodynamic studies were performed in the temperature range of 298–328 K. The Gibbs free energy value was calculated using the Gibbs–Helmholtz equation (Table 2). Using the van’t Hoff equation and from the equation describing the graphical evolution of ln *K_d_* = f(1/*T*), the standard variation of entropy Δ*S*^0^ and the standard variation of enthalpy Δ*H*^0^ can be calculated. The equilibrium constant is the ratio between the equilibrium adsorption capacity *q_e_* and the equilibrium concentration *C_e_*. The activation energy *E_a_* and the rate constant from the pseudo-second-order kinetic model *k*_2_ were calculated using the Arrhenius equation.

In order to establish the adsorption mechanism, the experimental data were fitted with OriginPro software (version 9.0.0), using three adsorption isotherms: Langmuir, Freundlich, and Sips (Table 3).

### 2.3. Fixed-Bed Column Adsorption Study

For the dynamic adsorption study of As(V) from aqueous solutions using the sludge obtained from the treatment of deep drinking water, a column with a diameter of 2 cm and a length of 30 cm was employed. The height of the material layer in the column was *H_layer_* = 10 cm. The total mass of material in the column was 10 g (5 g of UMIC and 5 g of sand).

To initiate the adsorption process, a solution containing 350 mg/L of As(V) was continuously introduced into the column using a peristaltic Heidolph SP quick pump (Heidolph Instruments GmbH & Co., Schwabach, Germany) at a flow rate of 10 mL/min. A volume of 3000 mL of water containing arsenic was passed through the column for 380 min. Sequential samples of 25 mL were collected, and the residual concentration of As(V) was analyzed using atomic absorption spectrophotometry.

Critical parameters for evaluating the effectiveness of the adsorbent material in a dynamic regime include the effluent flow rate within the column, the height of the fixed bed, and absorption time. These parameters play a significant role in characterizing the performance of the adsorption system [33,34].

Breakthrough curves are used to describe the dynamics of the adsorption process. These curves represent concentration plots that relate the initial concentration of As(V) to the duration of the process [35,36]. The shape and characteristics of these curves provide insight into the progression of the process, illustrating the saturation of the bed with the target substance.

To determine the adsorption mechanism in the dynamic regime and facilitate the design of the dynamic adsorption process, three models were utilized: the Adams–Bohart model, the Clark model, and the Thomas model (Table 4). The Adams–Bohart model focuses on describing the initial phase of the breakthrough curve, while the Clark model assumes a constant mass transfer zone and complete removal of adsorbate at the column’s end. The Clark model successfully works for other cases, including systems that have a variable mass transfer area. The Thomas model is widely employed to evaluate the performance of adsorption columns and establish breakthrough curves. It is frequently utilized for determining the adsorption capacity of the adsorbent material.

### 2.4. Statistical Analysis of Errors

To select the best model that fits the experimental data, a statistical analysis of errors is essential. This is because kinetic models and adsorption isotherms have different numbers of parameters, making it difficult to compare them using the same criteria. Furthermore, the determination coefficient (*R*^2^) is not a sufficient indicator of model fit. The following statistical equations (Table 5) are commonly used in error analysis for kinetic models and adsorption isotherms: residual sum of squares (*RSS*) and Bayesian information criterion (*BIC*). Based on these statistical tests, the model with the lowest *RSS* and *BIC* values is the best fitting model.

## 3. Results and Discussion

### 3.1. Adsorbent Characterization

Table 6 shows the chemical composition of the sludge resulting from the process of treating underground water for potable purposes. The studied sludge has a high iron content, recommended as a possible adsorbent material for the removal of arsenic from aqueous solutions.

**Figure 1:**
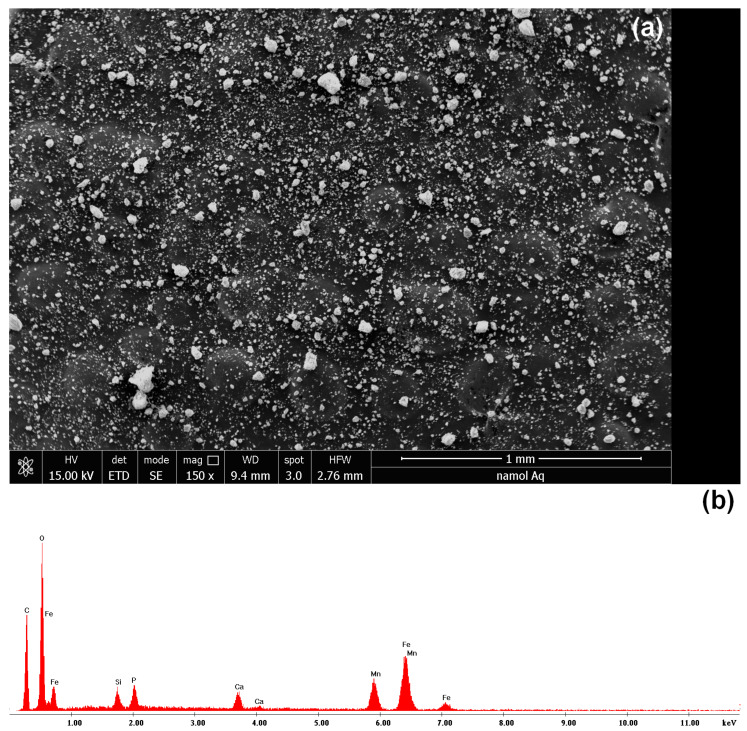
Scanning electron microscopy SEM (**a**) and energy-dispersive X-ray spectroscopy EDX (**b**) for adsorbent material (UMIC).

To highlight the morphology and the elemental composition of the studied material, the SEM image and the EDX spectrum are presented in Figure 1. Figure 1a reveals the distinctive granular architecture of the material, while Figure 1b unveils discernible peaks that correspond to the chemical elements comprising the material’s composition, namely iron (Fe), manganese (Mn), and calcium (Ca) in significant proportions. Furthermore, a minor presence of carbon (C) was observed, likely attributable to the carbon substrate utilized during the sample analysis. The Silicon (Si) present in smaller quantities is attributed to the natural fund (sand). The existence of oxygen (O) substantiates the occurrence of the cations primarily in oxide form.

**Figure 2:**
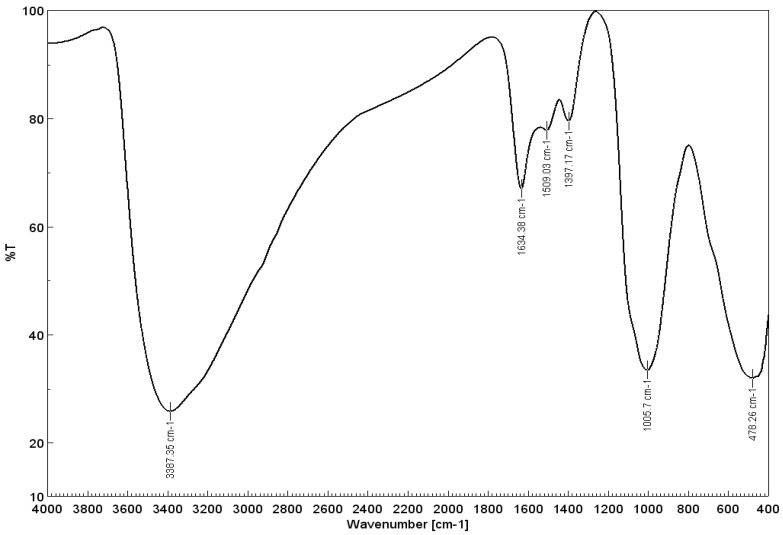
FT-IR spectrum recorded for the adsorbent material (UMIC).

FT-IR spectroscopy is a useful tool for identifying functional groups in a molecule because each specific chemical bond has a unique energy absorption band, and can obtain structural information about a complex such as iron or manganese oxy-hydroxides, as presented in Figure 2.

Within the spectral analysis, two discernible vibrational modes associated with the –OH group were observed at 3387 cm^−1^ and 1634 cm^−1^. These vibrations can be attributed to the bending motion of absorbed water molecules and the surface hydroxyl groups. Furthermore, the emergence of distinct peaks at 478 cm^−1^ correlated with the stretching vibrations corresponding to the Fe-O bond, indicating the presence of iron oxide-hydroxide compounds [40,41].

**Figure 3:**
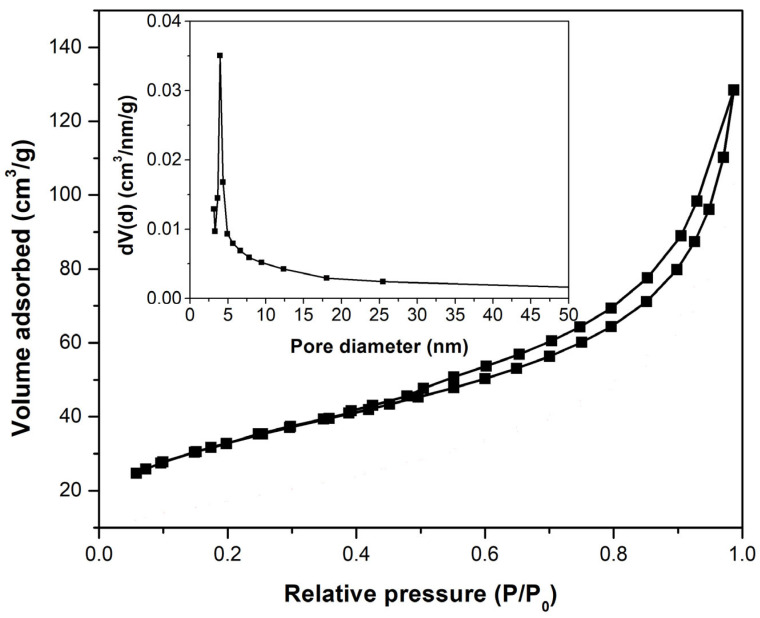
BET analysis of the adsorbent material (UMIC).

The material pore size and the specific surface area were determined using the BET method, after the samples were degassed in a vacuum for 12 h at 100 °C. In Figure 3, the N_2_ adsorption–desorption isotherms obtained by 44 points are presented. In the inset, the pore size distribution obtained by the BJH method is shown. For determining the surface area, the BET method was used. Figure 3 illustrates a type IVa isotherm with an H3 hysteresis specific for plate-like particles. The textural parameters From N_2_ obtained from the sorption isotherms are presented in Table 7 [42].

**Figure 4:**
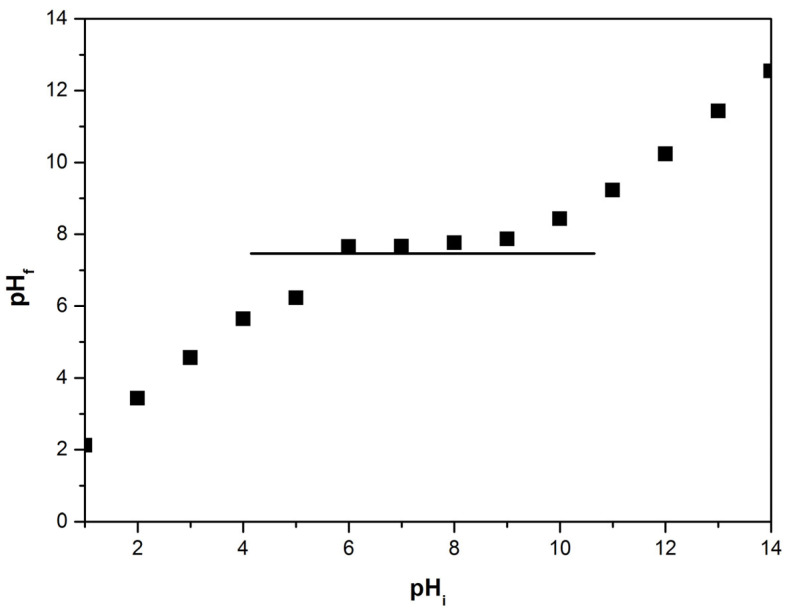
Determination of point of zero charge, pH_PZC_, for adsorbent material (UMIC).

The pH_PZC_ represents the pH at which the surface exhibits a neutral electric charge density, suggesting the likelihood of adsorption through the ion exchange mechanism. The determination of the material’s point of zero charge (pH_PZC_) serves as a valuable instrument to ascertain its surface charge properties, and provides insights into the adsorbate species that are prone to adsorption on its surface. The pH_PZC_ determination for the studied absorbent is presented in Figure 4. The pH_PZC_ value for using the sludge resulting from the treatment of groundwater is 7.65. At pH values lower than 7.65, the material’s surface predominantly carries a positive charge, while at higher pH values, it tends to exhibit a negative charge. It is noteworthy that the pH_PZC_ value falls within the pH range typically encountered in natural water systems. The primary adsorbing species for As(V) are neutral in nature, namely H_2_AsO_4_^−^ and HAsO_4_^2−^ [43].

### 3.2. Batch Adsorption Study

#### 3.2.1. pH Effect

**Figure 5:**
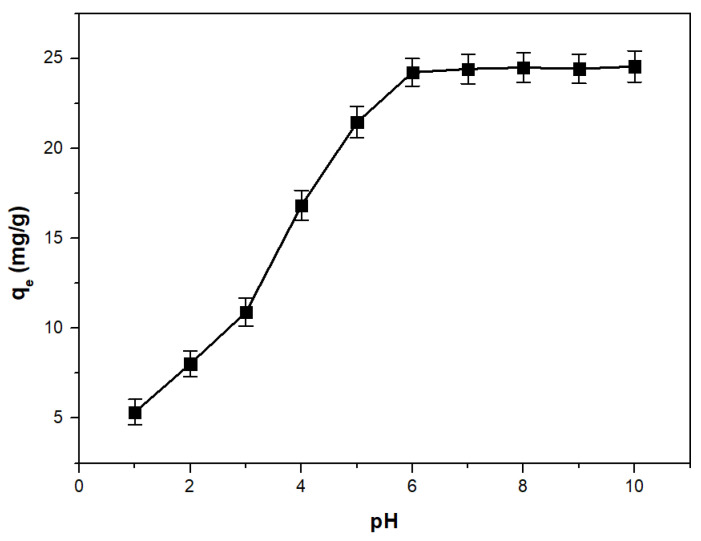
The pH effect on the adsorption capacity of the adsorbent material (UMIC) for arsenic adsorption.

The effectiveness of the adsorption process is highly dependent on the pH of the solution, as pH variations lead to changes in the degree of ionization of the adsorbed molecules and the properties of the adsorbent surface [44]. To identify the optimal conditions for the removal of As(V) from aqueous solutions, the adsorption capacity of the studied material was investigated in relation to the pH variation (Figure 5). The optimum pH for As(V) removal from solutions should be greater than 6. It can be found that the optimum pH for As(V) removal coincides with the pH_PZC_ of the material, and falls within the optimum pH range of drinking water.

#### 3.2.2. Contact Time and Temperature Effect

**Figure 6:**
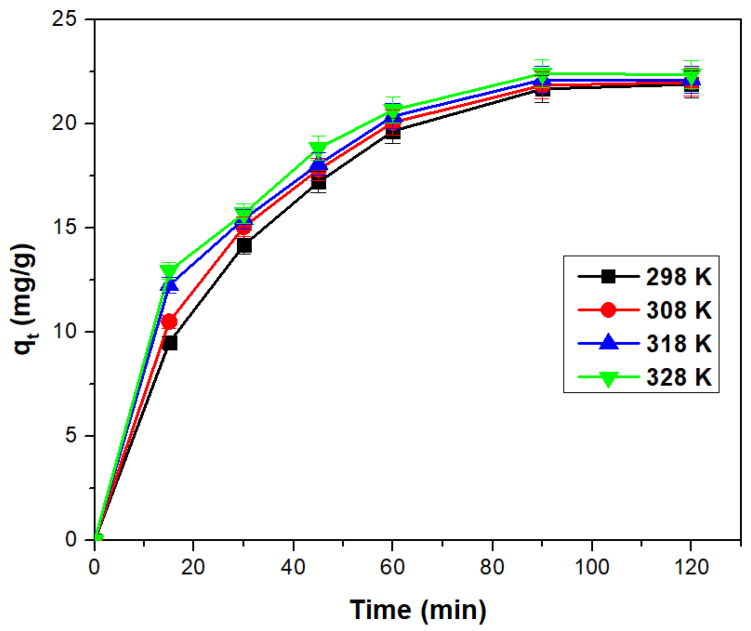
Contact time and temperature effect on the adsorption capacity of the adsorbent material (UMIC) for arsenic adsorption.

The contact time and temperature affect the adsorption process influencing the adsorption capacity of the material. Figure 6 shows the effect of the contact time upon the adsorption capacity of the studied material at four distinct temperatures. The experimental data illustrate the influence of contact time and temperature on the adsorption capacity, yielding a positive effect. It was observed that the adsorption capacity increases with prolonged contact time, reaching a point where further increases in contact time do not lead to a significant change in the adsorption capacity. Consequently, after 90 min, the adsorption capacity stabilizes at approximately 21 mg/g.

Regarding the temperature, an increase in temperature results in a slight increase in the adsorption capacity, but insignificantly, not justifying further studies at temperatures higher than 298 K. Moreover, considering the economic perspective, the benefits of increasing the temperature may not be justified.

#### 3.2.3. As(V) Initial Concentration Effect

In order to establish the adsorption process equilibrium of the As(V) on the studied adsorbent, it is very important to know what the maximum concentration of the adsorbant is.

**Figure 7:**
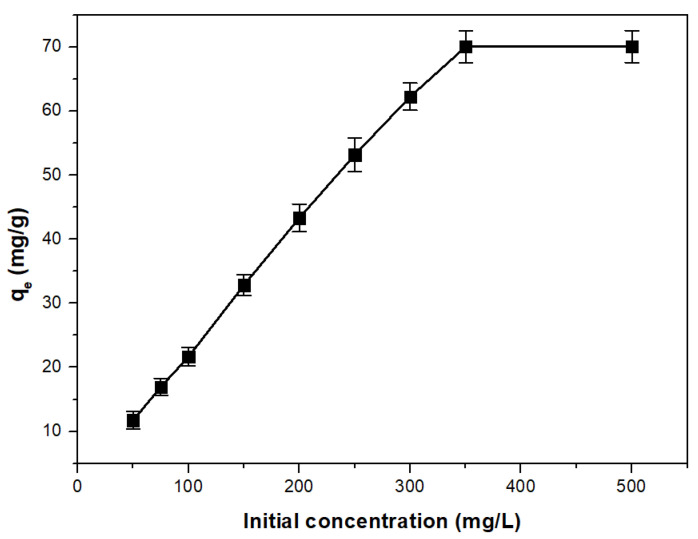
As(V) initial concentration effect on the adsorption capacity of the adsorbent material (UMIC) for arsenic adsorption.

The results we obtained illustrate that as the initial concentration of As(V) ions rises, the material’s adsorption capacity increases until it reaches saturation, at which point the adsorption capacity stabilizes (Figure 7). Consequently, the material’s maximum adsorption capacity is approximately 70.1 mg of As(V) per gram when the initial As(V) concentration is around 350 mg/L.

#### 3.2.4. Kinetic Study

**Figure 8:**
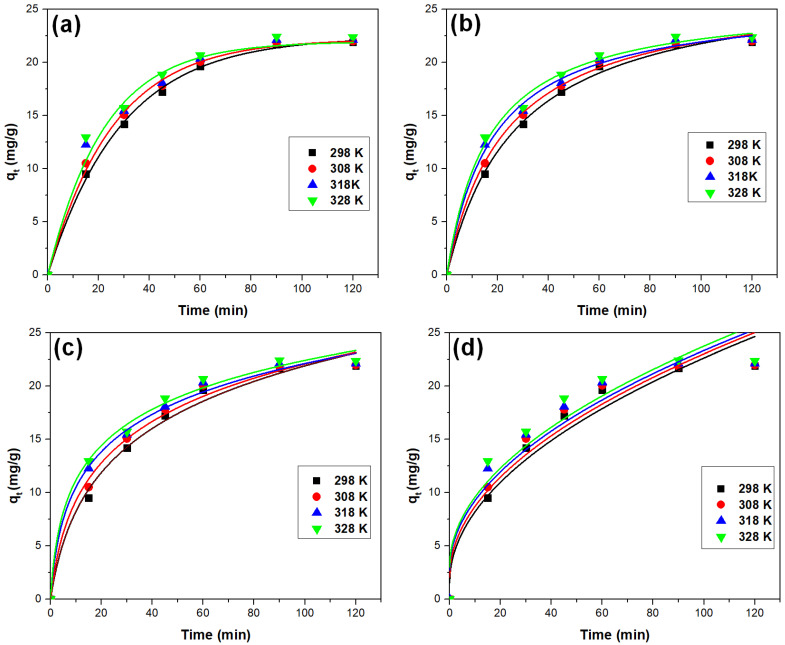
Kinetic studies: the fitted pseudo-order kinetic model (**a**), the pseudo-order kinetic model (**b**), the Elovich model (**c**), and intraparticle diffusion model (**d**).

The kinetics of the recovery process of As(V) by adsorption on the proposed material was studied using four kinetic models (Figure 8). The nonlinear forms of the two kinetic models were used to model the experimental data. The values of the rate constants and of the adsorption capacity calculated following the modeling are presented in Table 8. Furthermore, the values of the determination coefficient *R*^2^ are presented in the same table. The experimental data indicate an excellent fit with the pseudo-second-order kinetic model, confirmed by the value of the determination coefficient *R*^2^ that was between 0.9956 and 0.9970. In contrast, when modeling the data with the pseudo-first-order kinetic model, the determination coefficient deviated from 1. Additionally, the calculated *q_e,calc_* values based on the pseudo-second-order isotherm closely matched the experimental *q_e,exp_* values.

The temperature does have an influence on the parameters *k*_2_ and *q_e,calc_*, but this effect is not substantial. As a result, it is advisable to conduct the experiments at temperatures lower than 298 K to enhance the efficiency of the process.

#### 3.2.5. Thermodynamic Study

**Figure 9:**
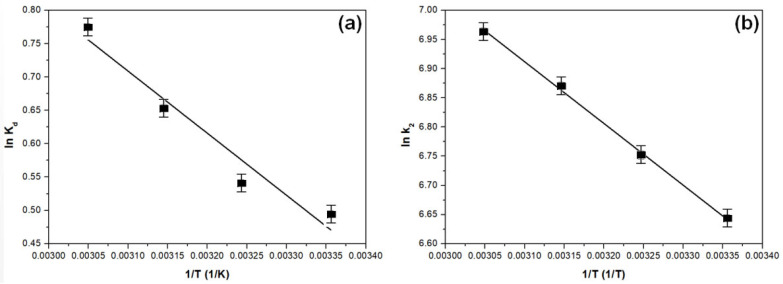
Thermodynamic studies: the fitted Van’t Hoff (**a**) and Arrhenius (**b**) equations.

The primary thermodynamic parameters, which encompass enthalpy (Δ*H*^0^), free energy (Δ*G*^0^), and entropy (Δ*S*^0^), were determined from the slope of the line established by the equation ln *K_d_* = f(1/*T*), as illustrated in Figure 9a. The values for Δ*G*^0^, Δ*H*^0^, and Δ*S*^0^ are presented in Table 9.

The activation energy (*E_a_*) value offers information about the adsorption process’s characteristics. It represents the minimum energy required by the reactants to transform into the reaction products, and can be determined from the relationship ln *k*_2_ = f(1/*T*).

Based on the experimental data shown in Figure 9b, the calculated activation energy is approximately 8.15 kJ/mol. Given that the activation energy for the recovery of As(V) through material adsorption is approximately 8 kJ/mol, it suggests that the adsorption process primarily exhibits physical characteristics [45].

#### 3.2.6. Equilibrium Study

**Figure 10:**
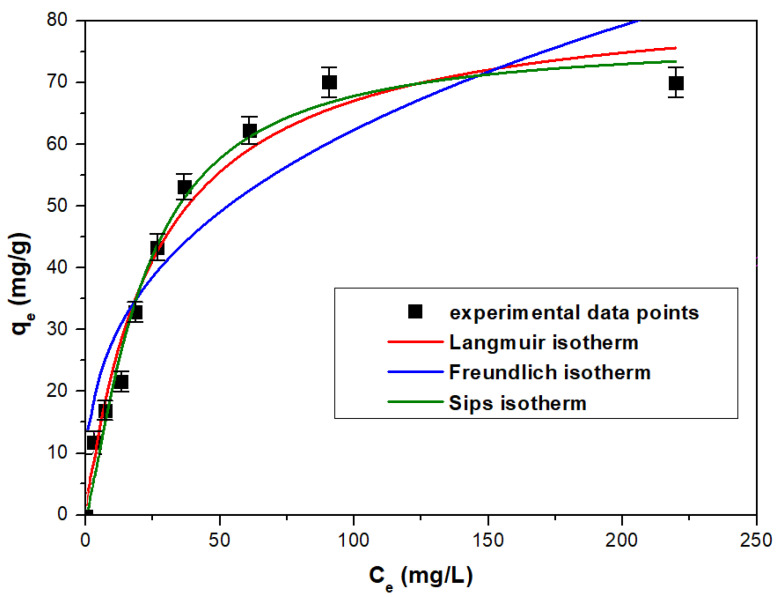
Equilibrium studies: the fitted equilibrium isotherms for modeling the experimental data.

The As(V) adsorption process mechanism on the studied material was established using the Langmuir, Freundlich, and Sips models. The adsorption isotherms, obtained using the graphical representation of *q_e_* = f(*C_e_*), are illustrated in Figure 10. The isotherms‘ specific parameters are presented in Table 10. According to the results of modeling the experimental data, the Sips isotherm proved to be the most suitable model for accurately representing the experimental data, with an *R*^2^ value of 0.9894, very close to the ideal value of 1. Furthermore, the maximum adsorption capacity calculated using the Sips isotherm, 76.8 mg/g, closely aligns with the experimentally obtained value of 70.1 mg/g (for an initial concentration of 350 mg As(V)/L).

Table 11 provides an overview of various materials documented in the literature that have been employed for the removal of As(V).

The material examined in this research, derived from the sludge generated during the treatment of drinking groundwater, proves to be an economical and environmentally sustainable option for effectively removing As(V) from aqueous solutions through static-mode adsorption.

### 3.3. Fixed-Bed Column Adsorption Study

#### 3.3.1. Mass Transfer Zone

Batch operations are straight forward for laboratory investigations Additionally, the data needed to design a pilot-scale fixed-bed system cannot be derived solely from adsorption isotherms obtained from batch-type experiments. Fixed-bed column adsorption offers several advantages, including ease of operation, high efficiency, and seamless scalability, from laboratory research to pilot or industrial setups [17,22].

In general, an effective arsenic adsorbent must meet the following specific criteria: (1) cost-effectiveness, (2) granular form, (3) high adsorption capacity and selectivity, (4) strong physical durability (resistance to disintegration in water), and (5) regenerability or reusability [61]. In order to fulfill the first and third requirements among all the materials studied, the unconventional option with iron content, namely, sludge derived from the treatment of deep drinking water, demonstrates substantial adsorption capacity in the removal of arsenic from water. The utilization of sludge offers notable advantages, both economically and in terms of environmental protection, as it repurposes waste from another technological process.

As the adsorbent is in powder form, it was mixed with sand to prevent column clogging. Previous studies have determined that the optimal sludge-to-sand ratio is 1:1, resulting in the highest adsorption capacity [62]. The fine particles of the sludge permeate the gaps between the sand grains, creating an extensive contact surface that promotes efficient adsorption. If a larger quantity of sludge is used, the column becomes clogged prematurely. Conversely, with an increased amount of sand, the mixture contains insufficient sludge (the active phase responsible for arsenic removal), leading to a decrease in the adsorption capacity.

Predicting the effluent breakthrough curve is an essential step in effectively designing a column adsorption process. The effect of the height of the adsorbent layer and the flow rate on the breakthrough curve for As(V) adsorption on the sludge:sand adsorbent mixture were investigated.

The empty bed contact time (EBCT) serves as a critical parameter that dictates the residence time during which the solution being treated comes into contact with the adsorbent. The EBCT can significantly influence adsorption, especially when the adsorption process depends on the duration of contact between the adsorbent and the adsorbate [63]. For our column, the EBCT fell within the range of 27 to 380 min when the sludge:sand ratio was 1:1.

The time required for penetration decreases as the amount of sludge increases. This, in turn, results in uneven distribution of the liquid within the column, which leads to a poor diffusion of the solution among the adsorbent particles.

In conclusion, the most effective performance of the sludge:sand mixture in the dynamic removal of As(V) from water is achieved when using a sludge:sand ratio of 1:1 and a flow rate of 10 mL/min.

#### 3.3.2. Adsorption Models for Column Study

In an adsorption column, phenomena such as axial dispersion, the external resistance of the film, and resistance to intraparticle diffusion may occur. To determine the adsorption mechanism of, and to effectively design the adsorption process in a dynamic environment, it is necessary to understand how the effluent’s residual concentration changes over time [64,65]. For this purpose, three models were used: the Bohart–Adams, the Thomas, and the Clark models.

**Figure 11:**
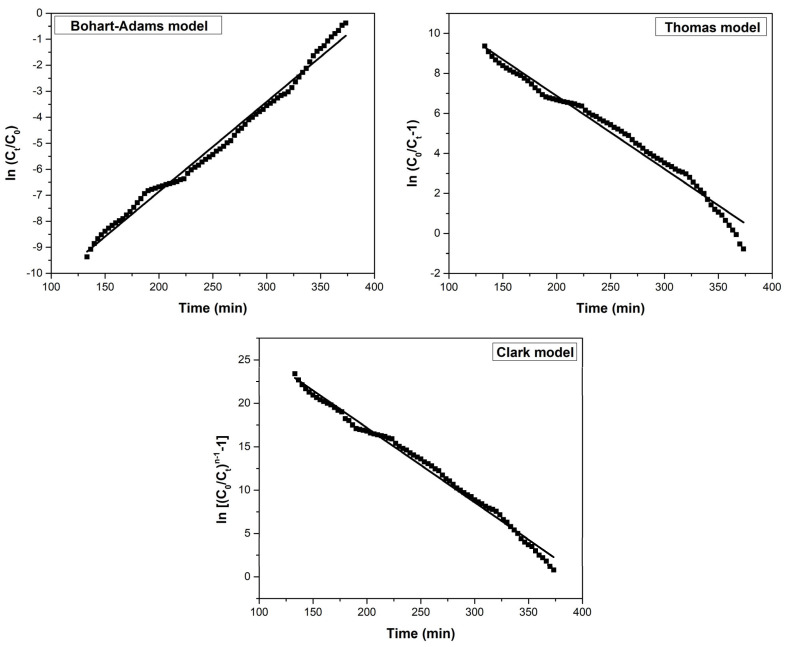
The tested column models’ plots for the adsorption of As(V).

The tested models‘ plots for the adsorption of As(V) and their specific parameters are illustrated in Table 12 and Figure 11. Analyzing the data, it can be found that the Clark model best characterizes the adsorption process. The model assumes that the shape of the mass transfer zone is constant, and that all of the adsorbents are removed at the end of the column [66]. The Clark model best described other adsorption processes of some metal ions in the column, such as Pd^2+^ on modified MgSiO_3_ [65] or Ag^+^ and Cu^2+^ on vermiculite [66].

## 4. Conclusions

Recognizing that water is an indispensable element for life and natural processes, our survival and economic activities rely entirely on this invaluable resource. Furthermore, water is a finite global resource, and despite extensive efforts and financial investments aimed at finding solutions, millions of people worldwide continue to consume arsenic-contaminated water daily. In response to the challenges posed by arsenic and the resulting interest from the scientific community, governmental bodies in affected regions, water industry corporations, as well as non-governmental organizations and agencies such as the World Health Organization and UNICEF, this study adopts an innovative approach. It explores the use of an unconventional material, specifically sludge generated from groundwater treatment for potable purposes, which proves to be a cost-effective and environmentally friendly option for efficiently removing arsenic from water. This study introduces a low-cost iron-rich waste material as a promising adsorbent for arsenic removal. The material is granular, has a high surface area (117 m^2^/g), is iron-rich (431.75 mg/kg dm), and has a pH_PZC_ of 7.65, which are optimal for arsenic adsorption. The material reaches its maximum adsorption capacity of 70.1 mg/g for an initial arsenic (V) concentration of 350 mg/L, with a maximum removal rate of 80.4% after 90 min at room temperature. The optimal pH for adsorption is pH > 6, and it is advisable to conduct studies within the pH range typical for drinking water. The pseudo-second-order kinetic model best fits the experimental data, as evidenced by its high *R*^2^ value and low *BIC*. The static adsorption process is spontaneous, endothermic, and primarily physical, as evidenced by the activation energy of ~8 kJ/mol. The Sips model best described the equilibrium data, with a maximum adsorption capacity of 70.1 mg/g As(V). The dynamic removal of arsenic(V) using a fixed-bed column containing varying mass ratios of our unconventional material/sludge and sand was studied. The most effective performance was achieved with a UMIC:sand ratio of 1:1 and a flow rate of 10 mL/min, which removed ~99.99% of arsenic(V) from 3000 mL of water in 380 min. To determine the adsorption mechanism and design the adsorption process in a dynamic environment, the authors used three models: the Adams–Bohart, Thomas, and Clark models. The Clark model best described the dynamic adsorption process. The proposed waste-derived adsorbent material is a promising alternative for arsenic removal from water, outperforming other similar low-cost iron-based materials.

## Figures and Tables

**Table 1:** Kinetic models used to describe the adsorption process.

Kinetic Model	Equation	References
Pseudo-first-order	$q_{t}=q_{e}\left(1-exp^{-k_{1}\times t}\right)$	[26]
Pseudo-second-order	$q_{t}=\frac{k_{2}\times t\times q_{e}{}^{2}}{1+k_{2}\times t\times q_{e}}$	[27]
Elovich	$q_{t}=\frac{1}{a}\ln\left(1+a\times b\times t\right)$	[28]
Intraparticle diffusion model	$q_{t}=k_{p}\sqrt{t}+C_{1}$	[28]

*q_t_* is the arsenic amount adsorbed at time *t*; *k*_1_, *k*_2_, and *k_p_* are the rate constants of pseudo-first-order, pseudo-second-order, and intraparticle diffusion models; *q_e_* are the theoretical values for the adsorption capacity; *a* is the desorption constant of the Elovich model; *b* is the initial velocity; and *C*_1_ is the boundary layer thickness.

**Table 2:** Equation used for thermodynamic study.

Equation Name	Equation	References
Gibbs–Helmholtz	$\Delta G^{0}=\Delta H^{0}-T\times \Delta S^{0}$	[29]
Van’t Hoff	$\ln K_{d}=\frac{\Delta S^{0}}{R}-\frac{\Delta H^{0}}{RT}$; $K_{d}=\frac{q_{e}}{C_{e}}$	
Arrhenius	$\ln k_{2}=\ln A-\frac{E_{a}}{R\times T}$	

Δ*G*^0^ is the free Gibbs energy standard variation, Δ*H*^0^ is the enthalpy standard variation, Δ*S*^0^ is the entropy standard variation, *T* is the absolute temperature, *K_d_* is the equilibrium constant, being calculated as ratio between the equilibrium adsorption capacity (*q_e_*) and the equilibrium concentration (*C_e_*), *k*_2_ is a speed constant, *A* is the Arrhenius constant, *E_a_* is the activation energy, and *R* is the ideal gas constant.

**Table 3:** Adsorption isotherms used to describe the adsorption process.

Isotherm	Equation	References
Langmuir	$q_{e}=\frac{q_{L}K_{L}C_{e}}{1+K_{L}C_{e}}$	[30]
Freundlich	$q_{e}=K_{F}C_{e}^{1/n_{F}}$	[31]
Sips	$q_{e}=\frac{q_{S}K_{S}C_{e}^{1/n_{S}}}{1+K_{S}C_{e}^{1/n_{S}}}$	[32]

*q_L_* and *q_S_* are the maximum absorption capacities; *K_L_*, *K_F_*, and *K_S_* are the Langmuir, Freundlich, and Sips isotherms constants; 1*/n_F_* is an empirical constant indicating the intensity of adsorption, and *n_S_* is the Sips isotherm exponent.

**Table 4:** Kinetic models used to describe the fixed-bed column adsorption.

Kinetic Model	Equation	References
Bohart–Adams	$\ln\left(\frac{C_{t}}{C_{0}}\right)=k_{BA}\cdot C_{0}\cdot t-k_{BA}\cdot N_{0}\cdot \frac{Z}{F}$	[37]
Thomas	$\ln\left(\frac{C_{0}}{C_{t}}-1\right)=\frac{k_{Th}\cdot q_{Th}\cdot m}{Q}-k_{Th}\cdot C_{0}\cdot t$	[38]
Clark	$\ln\left({\left(\frac{C_{t}}{C_{0}-C_{t}}\right)}^{n-1}\right)=\ln A-r\cdot t$	[39]

*C*_0_ is the influent concentration; *C_t_* is the solution concentration at time t in the effluent; *t* is time; *k_BA_* is the kinetic constant of the Bohart–Adams model; *F* is the linear velocity calculated by dividing the flow rate by the column section area; *Z* is the bed depth of column; *N*_0_ is the saturation concentration; *k_Th_* is the Thomas rate constant; *q_Th_* is the equilibrium compound uptake per g of the material; *m* is the mass of sorbent material; *Q* is the flow rate; *r* is the Clark model constant; and *A* is the Clark model constant.

**Table 5:** Statistical parameters used in error analysis for kinetic models and adsorption isotherms.

Parameter	Equation	Reference
Residual sum of squares	$RSS=\overset{n}{\underset{i}{\sum }}{\left(q_{e,exp}-q_{e,calc}\right)}^{2}$	[28]
Bayesian information criterion	$BIC=n\times \ln\left(\frac{RSS}{n}\right)+p\times \ln\left(n\right)$	

*q_e,exp_* is the experimental adsorption capacity; *q_e,calc_* is the theoretical adsorption capacity determined by the model; *n* is the number of experimental data; and *p* is the number of parameters estimated in the fit model.

**Table 6:** Sludge chemical composition.

Content (mg/kg d.m.)					
Fe	Mn	Mg	K	Ca	Na
431.75	22.77	3.20	0.30	36.07	1.05

**Table 7:** Textural parameters of samples.

Sample	Surface Area, BET, (m^2^/g)	BJH, Pore Size Distribution, Desorption, (nm)	Average Pore Size, (nm)	Total Pore Volume (cm^3^/g)
UMIC	117	3.974	6.814	1.99 × 10^−1^ for pores smaller than 145.9 nm

**Table 8:** Kinetic parameters for the adsorption of As(V) onto adsorbent (UMIC).

**Pseudo-First-Order**						
Temperature (K)	*q_e,exp_* (mg/g)	*k*_1_ (1/min)	*q_e,calc_* (mg/g)	*R* ^2^	*RSS*	*BIC*
298	21.66 ± 0.57	0.025 ± 0.002	14.95 ± 0.32	0.9594	1.19	−8.49
308	21.83 ± 0.62	0.025 ± 0.001	15.12 ± 0.41	0.9525	0.94	−10.28
318	22.10 ± 0.47	0.026 ± 0.002	16.06 ± 0.27	0.9356	4.24	0.39
328	22.40 ± 0.71	0.029 ± 0.003	17.71 ± 0.68	0.9282	4.92	1.43
**Pseudo-Second-Order**						
Temperature (K)	*q_e,exp_* (mg/g)	*k*_2_ (g/mg·min)	*q_e,calc_* (mg/g)	*R* ^2^	*RSS*	*BIC*
298	21.66 ± 0.57	767.70	25.73 ± 0.54	0.9956	0.58	−13.49
308	21.83 ± 0.62	854.64	25.77 ± 0.51	0.9970	0.92	−10.34
318	22.10 ± 0.47	964.08	26.45 ± 0.46	0.9967	1.63	−6.29
328	22.40 ± 0.71	1057.39	27.32 ± 0.34	0.9968	1.95	−5.02
**Elovich**						
Temperature (K)		*a* (g/mg)	*b* (mg/g∙min)	*R* ^2^	*RSS*	*BIC*
298		0.16 ± 0.02	2.14 ± 0.51	0.9891	3.71	−0.54
308		0.14 ± 0.01	1.59 ± 0.34	0.9881	3.38	−1.19
318		0.18 ± 0.02	3.28 ± 0.80	0.9924	2.31	−3.84
328		0.19 ± 0.02	4.01 ± 0.93	0.9917	2.58	−3.08
**Intraparticle Diffusion Model**						
Temperature (K)		*k_p_* (g/mg)	*C*_1_ (mg/g∙min)	*R* ^2^	*RSS*	*BIC*
298		2.37 ± 0.20	0.49 ± 0.05	0.9556	25.53	12.95
308		2.09 ± 0.17	1.98 ± 0.07	0.9417	21.81	11.84
318		2.05 ± 0.24	2.74 ± 0.14	0.9263	27.04	13.36
328		2.07 ± 0.31	3.03 ± 0.11	0.9150	31.94	14.51

**Table 9:** Thermodynamic parameters for adsorption of As(V) onto adsorbent (UMIC).

Δ*H*^0^ (J/mol)	Δ*S*^0^ (J/mol·K)	Δ*G*^0^ (kJ/mol)				*R* ^2^
7.73	29.87	298 K	308 K	318 K	328 K	0.9847
		−8.89	−9.19	−9.49	−9.79	

**Table 10:** Parameters of isotherm model for adsorption of As(V) onto unconventional material.

**Langmuir Isotherm**					
*q_m,exp_* (mg/g)	*K_L_* (L/mg)	*q_L_* (mg/g)	*R* ^2^	*RSS*	*BIC*
70.1	0.04	84.64	0.9633	132.9	30.47
**Freundlich Isotherm**					
*K_F_* (mg/g) (L/g)^1/*n*^	1/*n_F_*		*R* ^2^	*RSS*	*BIC*
12.64	0.35		0.8258	632.4	46.04
**Sips Isotherm**					
*K_S_*	*q_S_* (mg/g)	1/*n_S_*	*R* ^2^	*RSS*	*BIC*
0.38	76.8	0.11	0.9894	95.02	27.12

**Table 11:** Comparison of the studied adsorbent with other materials found in the literature that have been employed for the removal of As(V) in static mode.

Materials	Adsorption Capacity (mg/g)	References
Magnetic graphene oxide, MGOH	74.20	[46]
Graphene macroscopic composites, PGA/P-Fe_2_O_3_	76.72	[47]
Char carbon	34.46	[48]
Activated carbon	30.48	[48]
TiO_2_	41.00	[49]
Iron-containing material obtained by iron oxalate calcination, Fe^II^(COO)_2_·2H_2_O	0.49	[17]
Iron-containing material obtained by ferriammoniacal alum calcination, Fe^III^(NH_4_)(SO_4_)_2_·12H_2_O	0.53	[17]
Iron-containing material obtained by Mohr’s salt calcination, Fe^II^(NH_4_)_2_(SO_4_)_2_·6H_2_O	0.51	[17]
Fe(III)-loaded Amberlite XAD7-impregnated resin containing di(2-ethylhexyl) phosphoric acid, XAD-7-DEHPA-Fe impregnated by the dry method	0.176	[50]
Fe(III)-loaded Amberlite XAD8-impregnated resin containing di(2-ethylhexyl) phosphoric acid, XAD-8-DEHPA-Fe	0.226	[51]
Fe(III)-loaded Amberlite IR 120 (Na)-impregnated resin containing di(2-ethylhexyl) phosphoric acid,IR-120 (Na)-DEHPA-Fe	0.22	[52]
Fe(III)-loaded Amberlite XAD7-impregnated resin containing di(2-ethylhexyl) phosphoric acid and tri-n-octylphosphine oxide, XAD-7-DEHPA-TOPO-Fe	0.355	[53]
Fe(III)-loaded Amberlite XAD7-impregnated resin containing di(2-ethylhexyl) phosphoric acid and triphenylphosphine oxide, XAD-7-DEHPA-TPPO-Fe	0.298	[53]
Iron oxide-coated cement	0.670	[22]
Laterite	0.51	[54]
Chitosan Alginate Fe–sludge Beds (CAFBs) strengthened with manganese sludge	21.90	[55]
Iron oxide amended with rice husk	82.00	[56]
Coconut coir pith anion exchanger	2.36	[57]
Waste residues containing Fe and Mn oxides	132.0	[58]
Synthetic akaganeite (β-FeO(OH))	110.0	[59]
Chitosan–graphene oxide/gadolinium composite	252.1	[60]
**UMIC**	**70.1**	**This study**

**Table 12:** The tested models for the adsorption of As(V) and their specific parameters.

Column Adsorption Parameters Specifications					
Bohart–Adams model	*k_BA_* (L/mg·min)	*N*_0_ (mg/L)	*R* ^2^	*RSS*	*BIC*
	9.88 × 10^−5^	1477	0.9895	0.36	−234
Thomas model	*k_Th_* (L/mg·min)	*q_Th_* (mg/g)	*R* ^2^	*RSS*	*BIC*
	1.04 × 10^−4^	45,224	0.9770	0.55	−203
Clark model	*r* (1/min)	*A*	*R* ^2^	*RSS*	*BIC*
	0.086	2.71	0.9910	0.20	−276

## Data Availability

All of the experimental data obtained are presented in the forms of tables and/or figures in the article.
